# Optimization of Gas Composition Used in Plasma Chemical Vaporization Machining for Figuring of Reaction-Sintered Silicon Carbide with Low Surface Roughness

**DOI:** 10.1038/s41598-018-20849-5

**Published:** 2018-02-05

**Authors:** Rongyan Sun, Xu Yang, Yuji Ohkubo, Katsuyoshi Endo, Kazuya Yamamura

**Affiliations:** 1 Division of Precision Science & Technology and Applied Physics, Graduate School of Engineering, Osaka University, 2-1 Yamadaoka, Suita, Osaka, 565-0871 Japan; 2 Research Center for Ultra-precision Science and Technology, Graduate School of Engineering, Osaka University, 2-1 Yamadaoka, Suita, Osaka, 565-0871 Japan

## Abstract

In recent years, reaction-sintered silicon carbide (RS-SiC) has been of interest in many engineering fields because of its excellent properties, such as its light weight, high rigidity, high heat conductance and low coefficient of thermal expansion. However, RS-SiC is difficult to machine owing to its high hardness and chemical inertness and because it contains multiple components. To overcome the problem of the poor machinability of RS-SiC in conventional machining, the application of atmospheric-pressure plasma chemical vaporization machining (AP-PCVM) to RS-SiC was proposed. As a highly efficient and damage-free figuring technique, AP-PCVM has been widely applied for the figuring of single-component materials, such as Si, SiC, quartz crystal wafers, and so forth. However, it has not been applied to RS-SiC since it is composed of multiple components. In this study, we investigated the AP-PCVM etching characteristics for RS-SiC by optimizing the gas composition. It was found that the different etching rates of the different components led to a large surface roughness. A smooth surface was obtained by applying the optimum gas composition, for which the etching rate of the Si component was equal to that of the SiC component.

## Introduction

Reaction-sintered silicon carbide (RS-SiC) is very promising material for parts in equipment used for optical mirror devices in space telescope systems, molds for optical components, semiconductor and liquid crystal display (LCD) manufacturing and various production facilities because of its excellent properties, such as light weight, high rigidity, high thermal conductivity and low thermal expansion coefficient. Compared with traditionally used tungsten carbide (WC), RS-SiC has higher hardness, meaning that it has strong resistance to wear due to abrasion as well as the formation of scratches and subsurface damage (SSD). It also has high durability against high-temperature oxidation, making it suitable for use in long-life applications. Moreover, RS-SiC has high thermal conductivity and a low thermal expansion coefficient, therefore the likelihood of shape failures caused by nonuniformity of the temperature distribution in the molding process is very small^1–5^.

However, as the main component of RS-SiC is silicon carbide, RS-SiC is difficult to machine because of its high hardness and chemical inertness^6–13^. In addition, RS-SiC contains multiple components such as SiC and Si. Owing to the difference in the chemical and mechanical properties of SiC and Si grains, it is difficult to form an objective shape with an ultrasmooth surface. Conventional techniques such as turning and grinding using diamond are used to form a shape with high precision, high efficiency and low cost. However, scratches and an SSD layer are inevitably formed on the machined surface. As a highly efficient and damage-free figuring technique, atmospheric-pressure plasma chemical vaporization machining (AP-PCVM) was proposed^14,15^. AP-PCVM is an ultraprecision figuring technique that uses F radicals to change the surface of substrates to a volatile reaction product to form an objective shape. Since AP-PCVM is a noncontact chemical figuring technique that does not apply a mechanical load to substrates, an SSD layer is not formed in the removal process. In conventional low-pressure plasma processing, the material is etched by the reaction with chemically active radicals formed in a glow discharge, and the entire surface of the sample is exposed to the plasma and modified. In the application of pattern definition in integrated circuit (IC) fabrication, the etching of a photoresist mask layer on underlying layers is necessary. The material to be removed must be etched significantly faster than the underlying material. The underlying layer serves as a partial etchstop so that the etching layer can be etched throughout the sample^16–18^. In contrast, in the atmospheric-pressure plasma chemical vaporization machining (AP-PCVM) proposed in this study, local dry chemical etching is performed instead of full-surface etching. Thus, we can use AP-PCVM to achieve local processing without modifying the other locations and plasma-etch-resistant masks are not necessary. By controlling the dwell time, AP-PCVM enables the plasma figuring of freeform surfaces without a mask. AP-PCVM has been widely applied for the figuring of X-ray mirrors made of Si and for the thickness correction of quartz crystal wafers, and nanometer-order shape accuracy and thickness uniformity have been achieved^14,15^. Although AP-PCVM has been successfully applied for the figuring of single-component materials, there have been no reports of its application to multicomponent materials. In this study, experiments were conducted to investigate the optimal oxygen ratio in the gases used in AP-PCVM to obtain RS-SiC with a smoothly etched surface.

**Figure 1:**
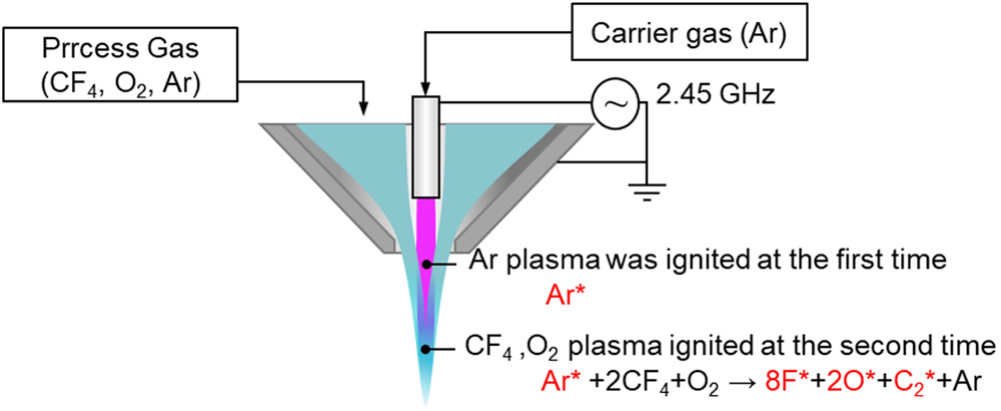
Schematic view of the plasma generator.

Figure 1 shows a schematic view of the plasma generator, which was installed on a computer numerical control (CNC) x-y-z-table. Argon gas was supplied as a carrier gas into the ceramic tube installed at the center of the plasma generator, which was surrounded by a cavity resonator. A microwave electric field with a frequency of 2.45 GHz was applied to generate an electric field whose maximum intensity was near the tip of the ceramic tube, where the argon atoms were ionized and atomic radicals were generated. The argon plasma was ignited at the first time. Simultaneously, argon, CF_4_ and O_2_ were supplied as the process gases from another gas inlet offset from the center. At this time, collisions occurred between argon in the active state originating from the center of the plasma generator and argon, CF_4_ and O_2_ in the ground state. As a result of the collisions, F radicals and O radicals, which contributed to etching, were generated at the second time. The reason why the CF_4_ was ionized at the second time but not ionized directly by the microwave electric field is that the F radicals are very reactive, and would have corroded the ceramic tube, decreasing its lifetime, and contamination due to the precipitation of substances originating from the etched ceramic tube would have interfered with the etching process. The argon gas flow rate was controlled by a flow control valve, and the CF_4_ and O_2_ gas flow rates were controlled by commercially available mass flow controllers (Tylan FC-770AC). The size of the RS-SiC substrate was 50 mm × 50 mm × 3 mm, and all the AP-PCVM experiments in this paper were conducted at room temperature without substrate heating. The temperature of the surface irradiated by plasma, which was measured by infrared thermography, was less than 50 °C.

## Results

### Effect of gas composition on etching rate

**Figure 2:**
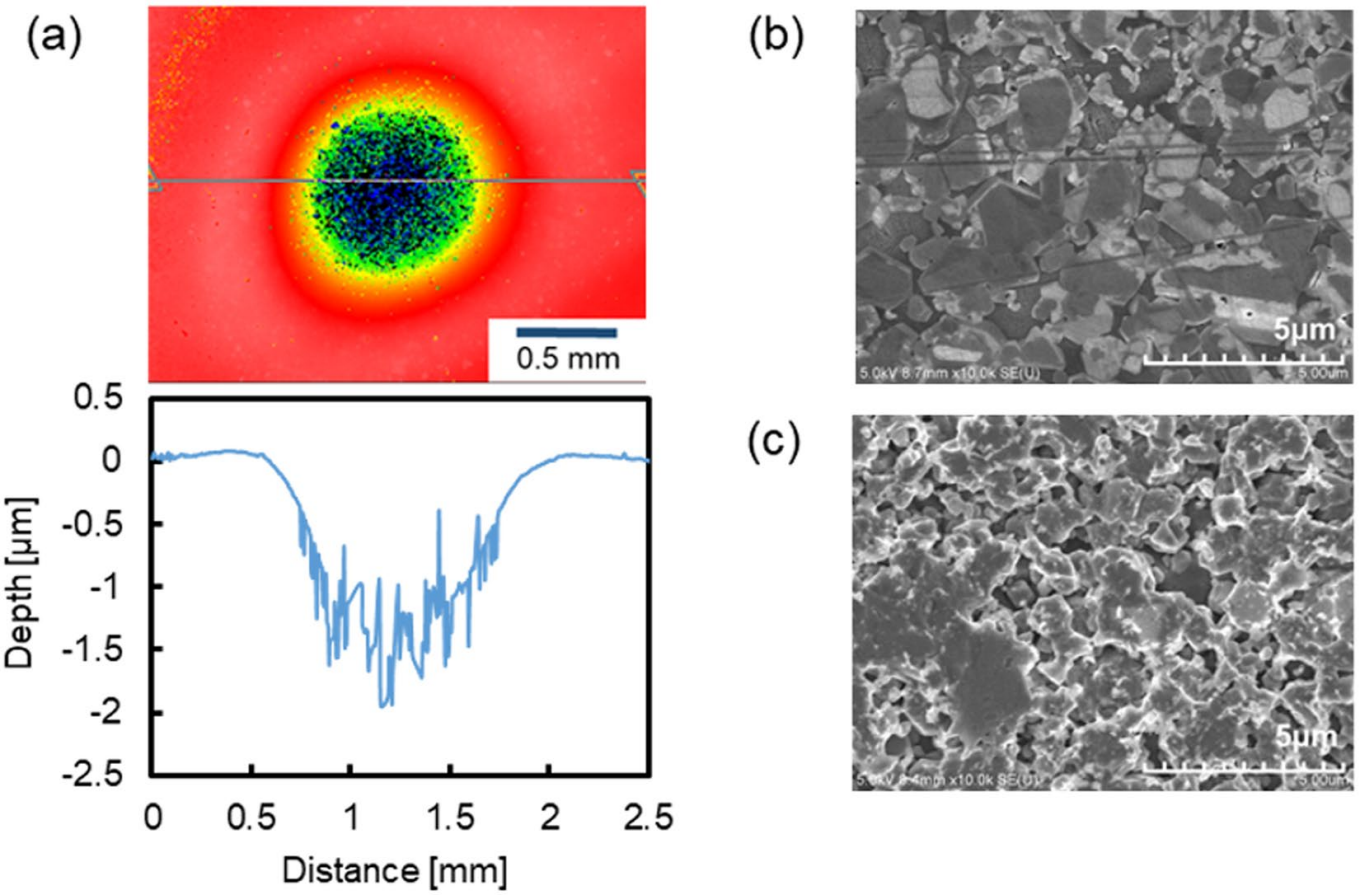
(**a**) SWLI image and cross section of a removal spot formed by AP-PCVM (oxygen fraction 50%), (**b**) SEM image of the surface prepared by diamond lapping, (**c**) SEM image of the center of the removal spot.

We first investigated the etching characteristics of RS-SiC. The carrier gas was Ar (500 sccm) and the process gases were Ar (1000 sccm), CF_4_ (50 sccm) and O_2_ (50 sccm). The working gap between the tip of the nozzle and the sample surface, the microwave power and the machining time were 6.0 mm, 59 W and 60 s, respectively. Figure 2a shows a scanning white-light interferometer (SWLI) image of a removal spot formed by AP-PCVM as well as its cross section. It was found that near the center of the removal spot, the roughness of the surface became large. Figure 2b shows a scanning electron microscope (SEM) image of the surface of RS-SiC prepared by diamond lapping. It can be observed that the regions between the SiC grains are filled with Si and that many scratches were formed on the surface. Figure 2c shows a SEM image of the center of the removal spot processed by AP-PCVM. In contrast to the image in Fig. 2b, it can be seen that the regions between SiC grains have been etched away. It is assumed that the difference between the etching rates of Si and SiC led to the large surface roughness. Thus, to obtain RS-SiC with a smoothly etched surface, it is essential to optimize the gas composition to make the etching rate of SiC equal to that of Si. A study on the relationship between the oxygen ratio in a process gas and the etching rate has been reported by Mogab *et al*. for a low-pressure CF_4_-O_2_ plasma etching process^19^. Their results showed that the addition of O_2_ to a CF_4_ plasma markedly increases the optical emission intensity from atomic fluorine, but the etching rate is not strictly proportional to the density of F radicals because of the competition between F radicals and O radicals for active Si surface sites.

**Figure 3:**
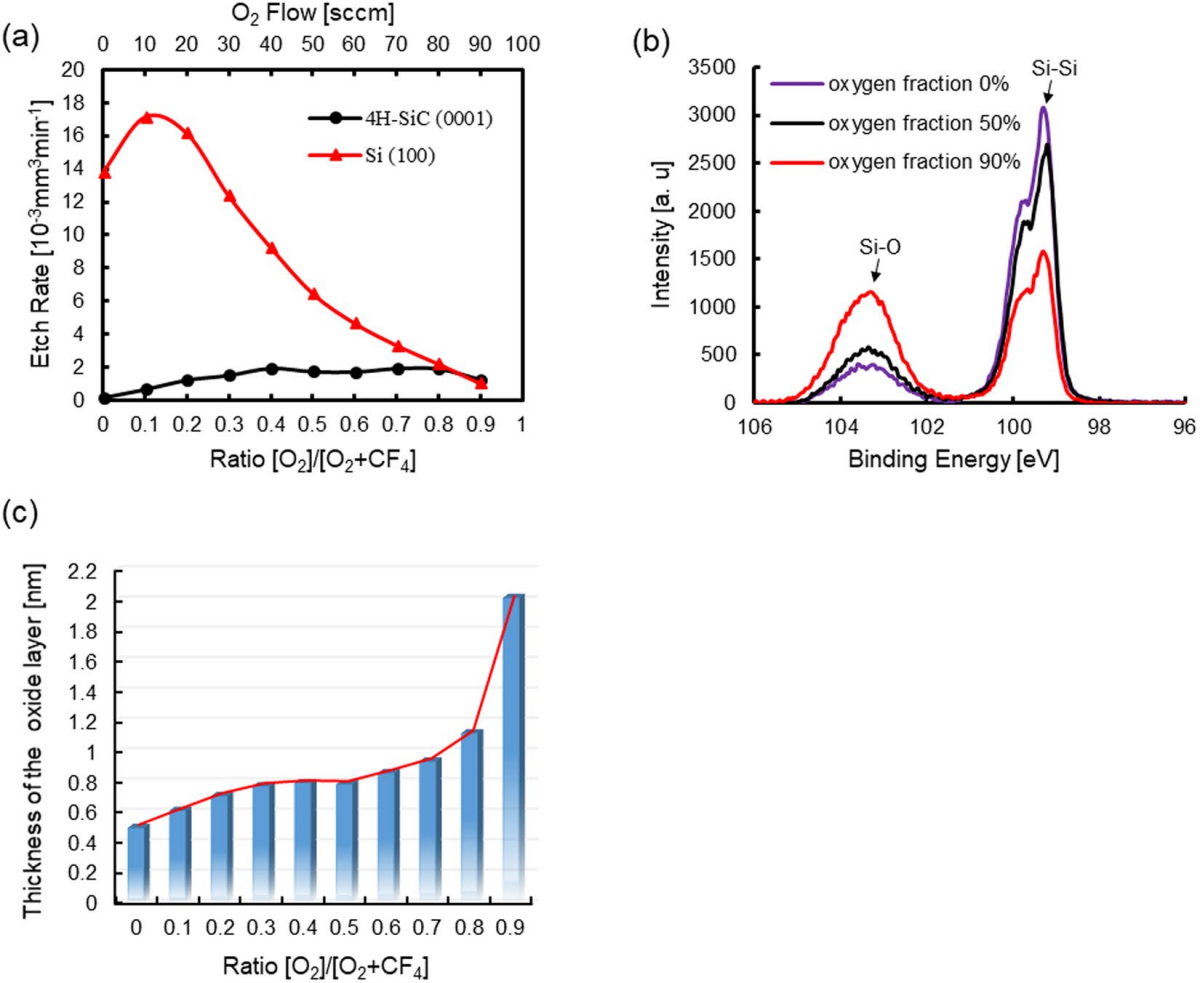
(**a**) Etching rates of Si (100) and 4H-SiC (0001) for different oxygen fractions, (**b**) Si2p-XPS spectra of Si (100) etched by AP-PCVM for oxygen fractions of 0%, 50% and 90%, (**c**) thickness of oxide layer on Si (100) surface for different oxygen fractions.

To investigate the AP-PCVM etching characteristics and find the process gas composition for which the etching rate of SiC is equal to that of Si, a similar experiment was conducted on 4H-SiC (0001) and Si (100) substrates using different [O_2_/(O_2_ + CF_4_)] ratios. The carrier gas was Ar (500 sccm) and the process gases were Ar (1000 sccm), CF_4_ (100-10 sccm) and O_2_ (0–90 sccm). The working gap was 6.0 mm and the machining time was 60 s. The obtained etching rates are shown in Fig. 3a. The experimental results we obtained were similar to those described in the papers of Yin^20^ and Mogab *et al*.^19^. The etching rate of Si (100) generally increased at low oxygen fractions and the maximum etching rate was obtained at an O_2_ concentration of about 10%. Then the etching rate decreased as the oxygen fraction further increased. However, the effect of oxygen addition on the etching rate of 4H-SiC (0001) was not as significant as that on Si (100). The 4H-SiC (0001) etching rate exhibited a much less marked peak than Si (100) at an O_2_ concentration of 40%. Since the bond dissociation energy of Si-Si (310 kJ/mol) is lower than that of Si-C (447 kJ/mol)^21^, it is considered that the lower bond dissociation energy of Si-Si led to a higher etching rate of Si (100) than that of 4H-SiC (0001) under the same conditions. To investigate the possible surface reactions, X-ray photoelectron spectroscopy (XPS) measurement of the surfaces etched by AP-PCVM with different [O_2_/(O_2_ + CF_4_)] ratios was conducted. Since oxygen is present in the atmosphere, although no oxygen was initially added to the process gases, a Si-O peak (103.5 eV) was observed on the surface as shown in Fig. 3b. The Si-O peak became stronger as the oxygen fraction increased. We calculated the thickness of the oxide layer from the ratio of the Si-O peak and Si-Si peak intensities using the oxide thickness determination equation^22–25^. As shown in Fig. 3c, with increasing oxygen fraction in the process gases, the thickness of the residual oxide layer on Si (100) also increased. Similar plasma simulation results have also been reported by Knizikevicius^26^. This means that as the oxygen fraction increased, the competition between the adsorption of oxygen and the etching by active species inhibited the etching process and led to a decrease in the etching rate at a high oxygen fraction. Despite O radicals being dominant owing to the high oxygen fraction, since the Gibbs free energy of SiF_4_ (−1573 kJ/mol) is lower than that of SiO_2_ (−853 kJ/mol), although the middle of the reaction process produced SiO_2_, the final reaction product was SiF_4_ rather than SiO_2_^27^. Thus, the removal process in AP-PCVM is not a simply an etching process but involves the competition between oxidation and etching processes. In addition, no Si-O peak was observed in the case of 4H-SiC (0001) at low oxygen fractions. Only for an oxygen fraction of 90% was an oxide layer observed, which had a thickness of 1.05 nm.

### Effect of gas composition on surface roughness of RS-SiC

**Figure 4:**
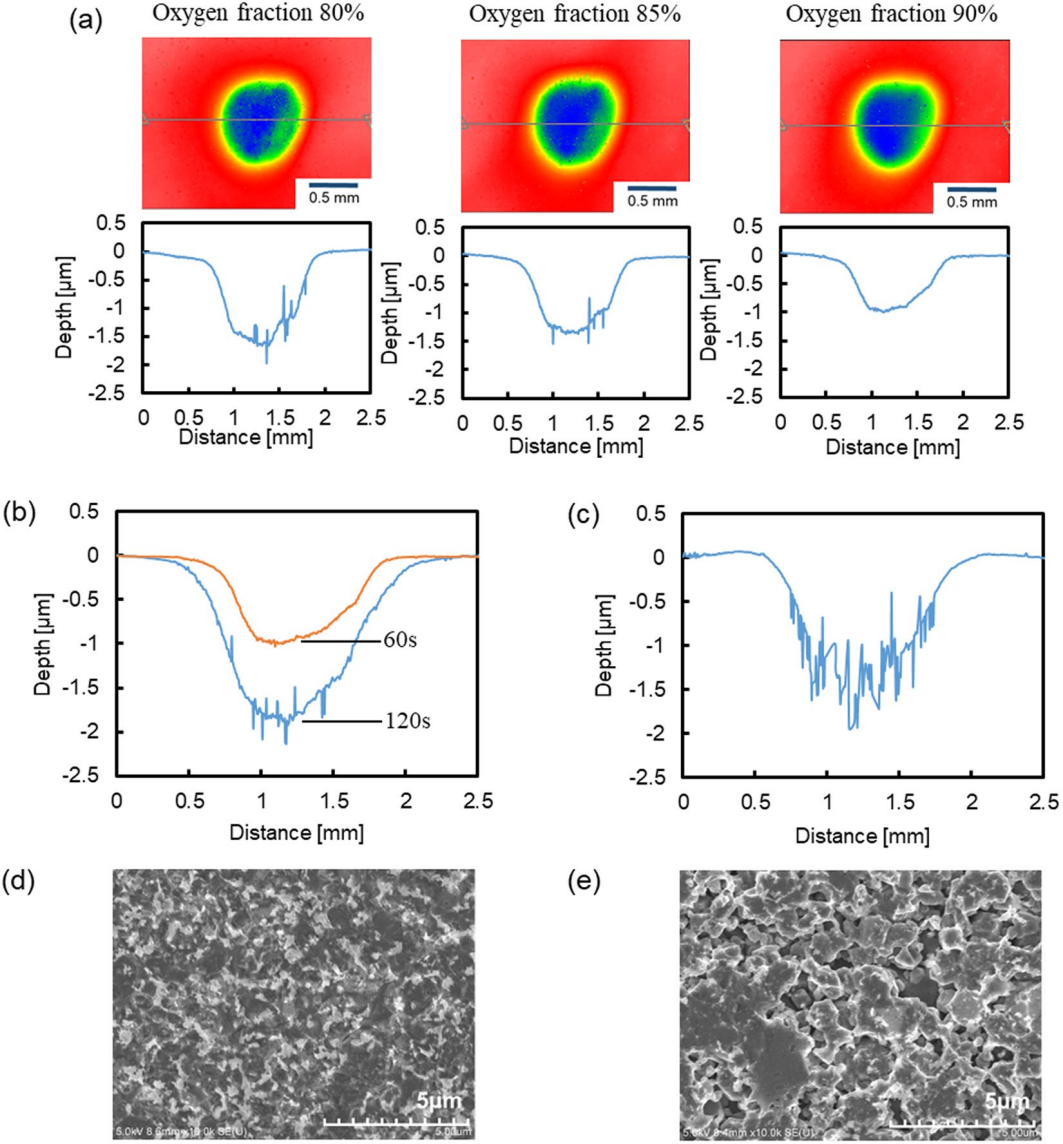
(**a**) SWLI images and cross sections of removal spots for oxygen fractions of 80%, 85% and 90%, (**b**) cross sections of removal spots etched with an oxygen fraction of 90% for 60 and 120 s, (**c**) cross section of removal spot etched with an oxygen fraction of 50% for 60 s, (**d**) SEM image of center of removal spot etched with an oxygen fraction of 90% for 120 s, (**e**) SEM image of center of removal spot etched with an oxygen fraction of 50% for 60 s.

In this study, we found that the gas composition for which the etching rate of Si (100) coincided with that of 4H-SiC (0001) had an oxygen fraction of between 80% and 90%. We already know that the reason for the large surface roughness of RS-SiC in AP-PCVM is the difference between the etching rates of Si and SiC. Thus, we consider that a smooth surface can be obtained when the etching rate of Si coincides with that of SiC. To find the optimum gas composition, experiments were conducted on RS-SiC with different oxygen fractions (80%, 85%, 90%). The carrier gas was Ar (500 sccm) and the process gases were Ar (1000 sccm), CF_4_ (20, 15, 10 sccm) and O_2_ (80, 85, 90 sccm). The working gap was 6.0 mm and the etching time was 60 s. Figure 4a shows SWLI images and cross sections of the removal spots. A smooth surface was obtained with an oxygen fraction of 90% but the removal spot was shallow. It is possible that the surface roughness deteriorates as the depth of the removal spot increases. To investigate this hypothesis, another experiment was conducted on RS-SiC with an oxygen fraction of 90% for 120 s. Figure 4b shows cross sections of removal spots etched with an oxygen fraction of 90% for 60 and 120 s, and Fig. 4(c) shows the cross section of a removal spot etched with an oxygen fraction of 50% for 60 s. The figures showed that the surface roughness of the removal spot etched with an O_2_ fraction of 90% was lower than that of the surface etched with an O_2_ fraction of 50% even for the same removal depth. Figure 4d and e show SEM images of the center of the removal spots etched with oxygen fractions of 90% and 50%, respectively. As shown in Fig. 4e, since the etching rate of Si was higher than that of SiC, the regions between SiC became concave, leading to an increase in surface roughness. However, when the etching rate of Si was equal to that of SiC at an O_2_ fraction of 90%, as shown in Fig. 4d, a smooth surface was obtained.

### Raster scanning of RS-SiC surface for different oxygen fractions

**Figure 5:**
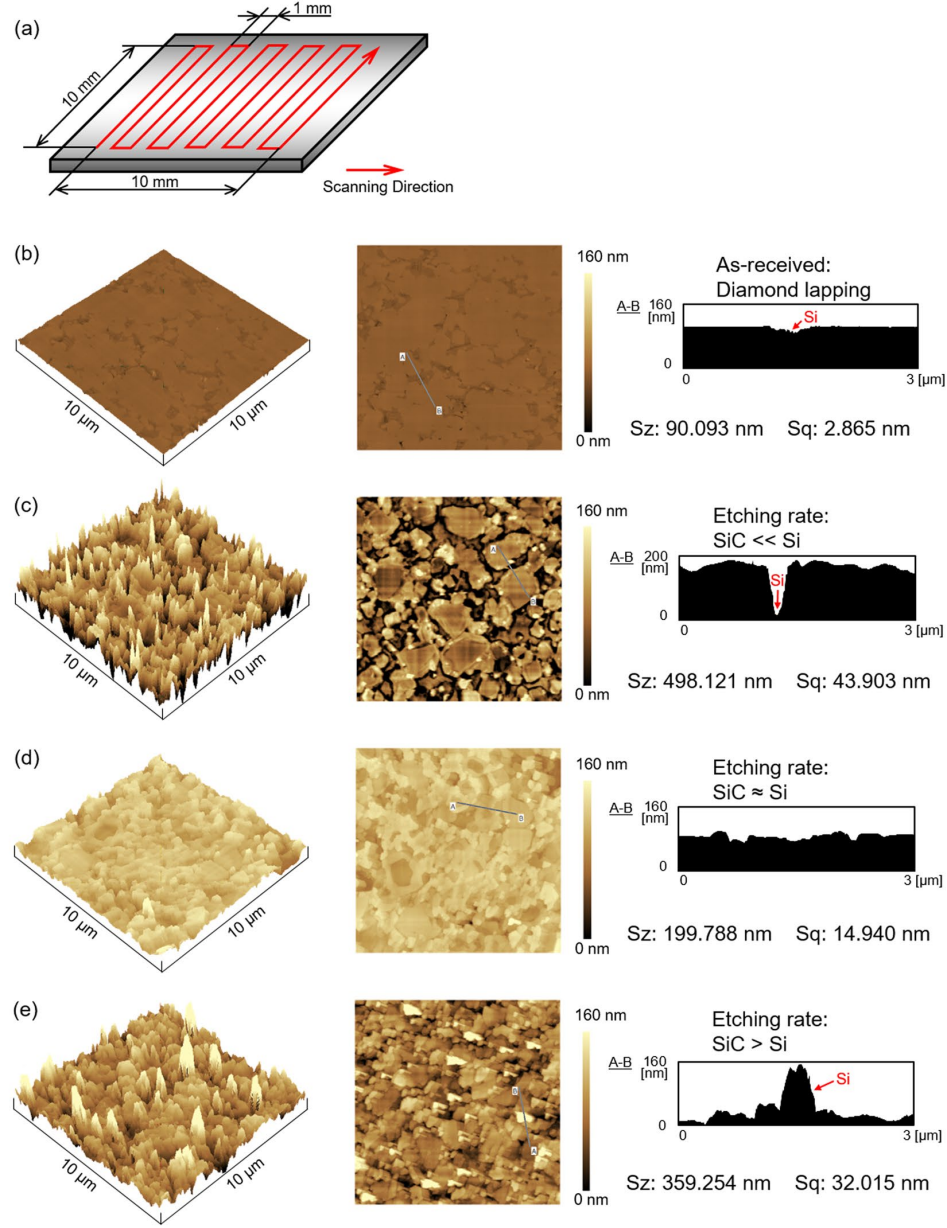
Raster scanning results for AP-PCVM. (**a**) Scanning path, (**b**) AFM image of original surface etched by diamond lapping before AP-PCVM, (**c**) AFM image of surface etched by AP-PCVM with oxygen fraction of 50%, (**d**) AFM image of surface etched by AP-PCVM with oxygen fraction of 90%, (**e**) AFM image of surface etched by AP-PCVM with oxygen fraction of 95%.

**Figure 6:**
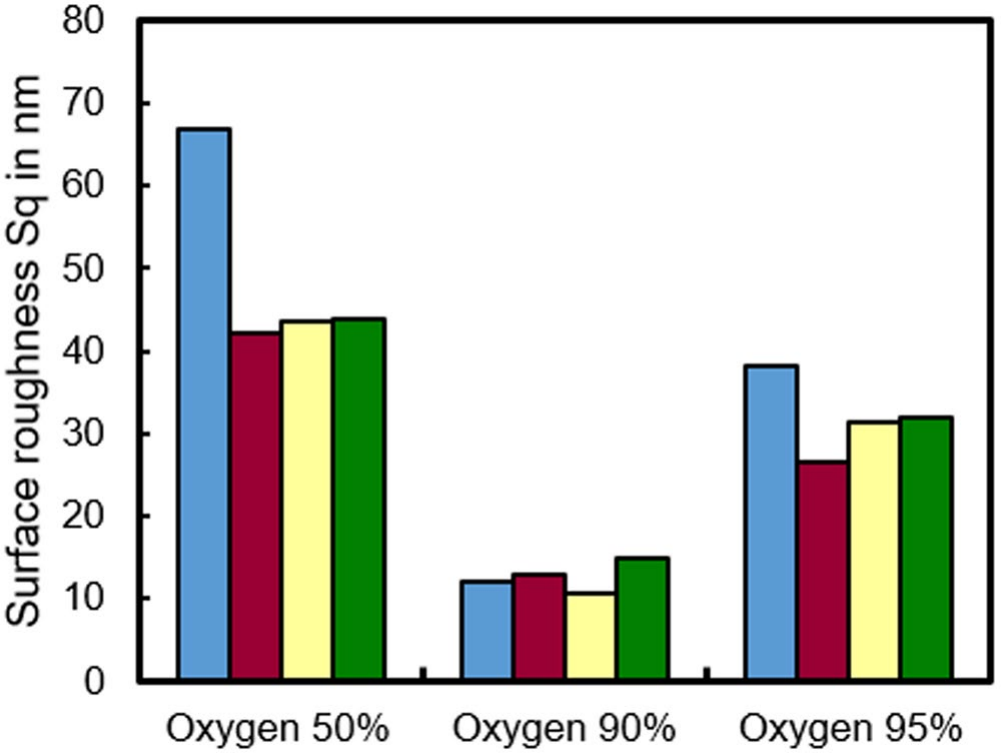
Variation in surface roughness of RS-SiC etched by raster scanning AP-PCVM with oxygen fractions of 50%, 90% and 95%.

In the previous section, we reported that a removal spot with a smooth surface can be obtained at an oxygen fraction of 90%. From the etching rate curve, we assume that the etching rate of Si (100) is greater than that of 4H-SiC (0001) when the oxygen fraction is less than 90% and that the etching rate of Si (100) is less than that of 4H-SiC (0001) when the oxygen fraction is more than 90%. In the actual application of AP-PCVM, since the volume of the removal spot increases with the processing time, the local removal volume at a certain position can be controlled via the dwelling time of the plasma jet during scanning^15^. A surface etching experiment was conducted to evaluate the roughness of surfaces etched by AP-PCVM with oxygen fractions of 50%, 90% and 95%. The carrier gas was Ar (500 sccm) and the process gases were Ar (1000 sccm), CF_4_ (50, 10, 5 sccm) and O_2_ (50, 90, 95 sccm). The working gap was 6.0 mm. The scanning path is shown in Fig. 5a. The size of the etched area in the AP-PCVM was 10 mm × 10 mm, and etching was performed in the raster scanning mode with a scan speed of 10 mm/min and a feed pitch of 1 mm. To ensure the same etching depth, the etching process was conducted twice with oxygen fractions of 90% and 95%. Figure 5b shows the initial surface before AP-PCVM, which was prepared by diamond lapping, whose roughness was measured by atomic force microscopy (AFM). Although the surface roughness was excellent, an SSD layer and scratches were formed as shown in Fig. 2b. Figure 5c shows an AFM image of the surface after etching with an oxygen fraction of 50%. Since the etching rate of Si was greater than that of SiC at this oxygen fraction, the regions between SiC were concave and the surface roughness deteriorated to 43.9 nm Sq. Figure 5d shows an AFM image of the surface after etching with an oxygen fraction of 90%. Since the etching rates of Si and SiC coincided at this oxygen fraction, a smooth surface was obtained. As AP-PCVM is an isotropic etching process, the surface became slightly rougher than the diamond lapped surface. Figure 5e shows an AFM image of the surface after etching with an oxygen fraction of 95%. Since the etching rate of Si was less than that of SiC, the regions between the SiC grains protruded. For this reason, the surface roughness deteriorated to 32.0 nm Sq. To demonstrate the reproducibility of the experiments, the experiment described above was conducted four times with three different oxygen fractions. Figure 6 shows the variation in the surface roughness of RS-SiC etched by raster scanning AP-PCVM with oxygen fractions of 50%, 90% and 95%. It was shown that the surface roughness was greatly decreased with an oxygen fraction of 90%.

## Discussion

We investigated the factors affecting the surface roughness in AP-PCVM. The addition of O_2_ to a CF_4_ plasma produced a marked increase in the optical emission intensity from electronically excited states of atomic fluorine^19^. However, as the oxygen fraction increased, competition between the adsorption of oxidation by O radicals and the etching by F radicals inhibited the etching process and led to a decrease in the etching rate at high oxygen fractions. Thus, the actual AP-PCVM process at high oxygen fractions is not simply an etching process but also involves competition between oxidation and etching processes. The possible chemical reactions that occur in the AP-PCVM of RS-SiC are as follows:

**Oxidation process:**

SiC + 4 O* → SiO_2_ + CO_2_↑

Si + 2 O* → SiO_2_

**Etching process:**

SiO_2_ + 4 F* → SiF_4_↑ + O_2_↑

SiC + 2 O* + 4 F* → SiF_4_↑ + CO_2_↑

Si + 4 F* → SiF_4_↑

**Figure 7:**
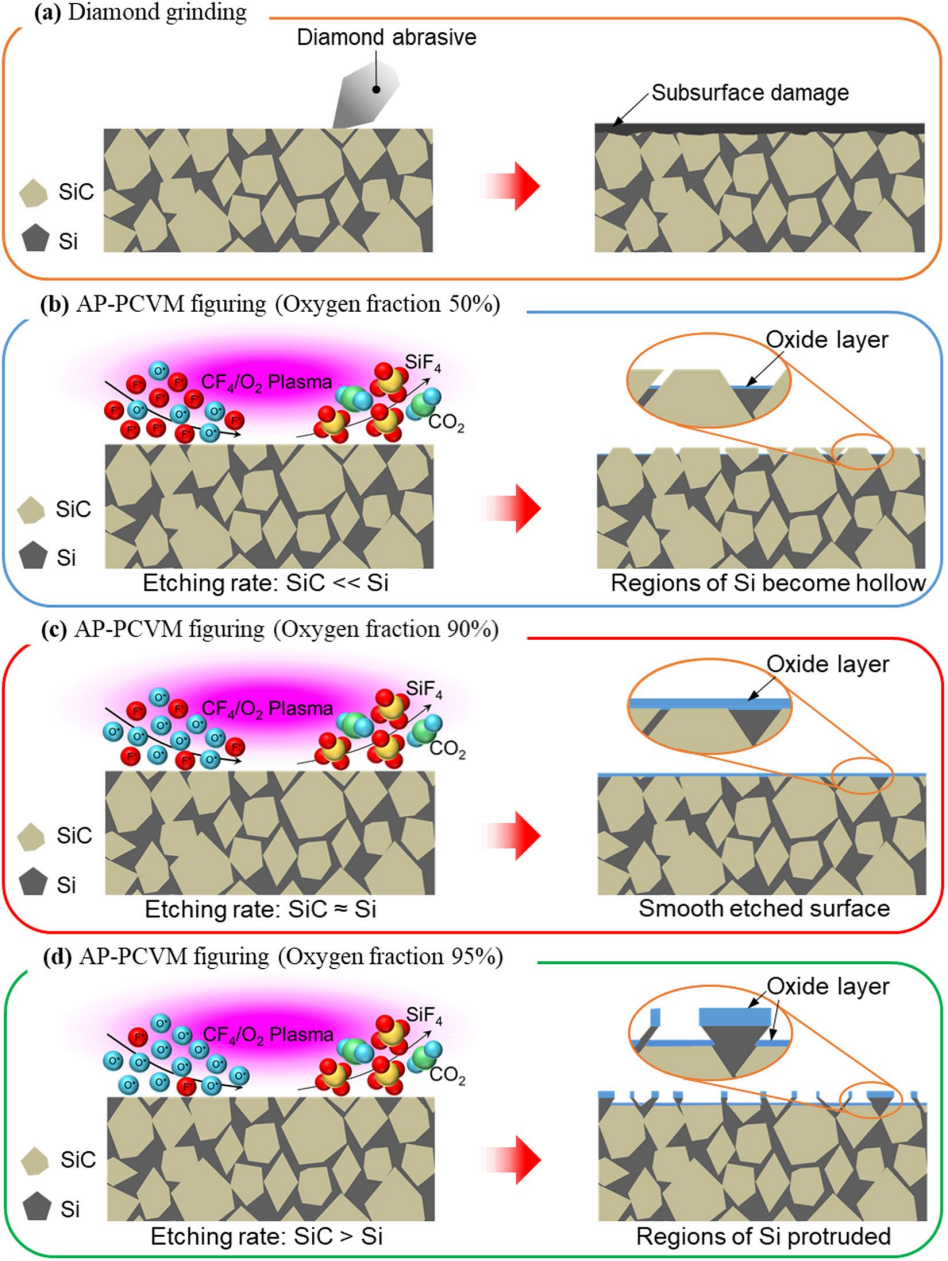
Models showing figuring process for RS-SiC. (**a**) Figuring by diamond grinding, (**b**) figuring by AP-PCVM (etching rate: SiC «Si), (**c**) figuring by AP-PCVM (etching rate: SiC ≈ Si), (**d**) figuring by AP-PCVM (etching rate: SiC > Si).

Upon optimizing the oxygen fraction of the process gases, the etching rates of both Si (100) and 4H-SiC (0001) changed. When the oxygen fraction was 90%, the etching rate of Si (100) coincided with that of 4H-SiC (0001). Thus, it is considered that the Si and SiC in RS-SiC were etched at the same rate at an oxygen fraction of 90%, resulting in a smooth surface. The models proposed for the AP-PCVM process on an RS-SiC surface at different oxygen fractions are shown in Fig. 7. As shown in Fig. 7a, turning and grinding with a diamond tool are used to realize figuring with high precision and high efficiency. However, scratches and an SSD layer are inevitably formed. Since AP-PCVM is a pure chemical non-contact process, scratches and an SSD are not formed. As shown in Fig. 7b, when the etching rate of Si was greater than that of SiC (oxygen fraction of 50%), the volume of removed Si was larger than that of SiC, causing the regions of Si to become hollow. As shown in Fig. 7d, when the etching rate of Si was less than that of SiC (oxygen fraction of 95%), the volume of removed Si was smaller than that of SiC; thus, the regions of Si protruded. Neither of the two conditions produced a smoothly etched surface. As shown in Fig. 7c, only when the etching rate of Si coincided with that of SiC (oxygen fraction of 90%) were the Si and SiC etched at the same rate and a smoothly etched surface was obtained. AP-PCVM has already been successfully applied to figuring single-component materials. Through this study, we have confirmed that for a multicomponent material, AP-PCVM with the optimum gas composition can also be used to obtain a smooth surface without forming SSD. Thus, it is expected that AP-PCVM can be applied to other multicomponent materials.

## Methods

### Materials

Reaction-sintered silicon carbide (RS-SiC) was fabricated by pressing infiltrated silicon and graphite into porous ceramic SiC in an electric furnace. The unreacted Si remained in the SiC. Thus, both a Si component and a SiC component existed in the RS-SiC. Because of its excellent properties, such as light weight (density lower than that of SiC), high rigidity, low thermal expansion and high thermal conductivity, RS-SiC has been of interest in many engineering fields.

### AP-PCVM experimental setup

The AP-PCVM experimental setup used in this study is laboratory-built. The main part of the setup comprises a CNC machine equipped with an x-y-z-table, a microwave power supply unit (Nagano. JRC, NJW-143), a gas supply system for plasma generation and a plasma generator (Nagano. JRC, NJW-144). By controlling the removal rate of the CNC machine to control the plasma dwelling time, the local removal volume at a certain position can be controlled via the dwelling time of the plasma jet during scanning.

### X-ray photoelectron spectroscopy

To examine the difference in the chemical components on AP-PCVM-etched surfaces for different oxygen fractions, angular-dependent X-ray photoelectron spectroscopy (XPS) measurements were conducted using a Quantum 2000 instrument (ULVAC-PHI) with Al-*K*α excitation (1486.6 eV) and a take-off angle of 30°. The area of X-ray irradiation was *Φ* = 100 μm, the pass energy was 23.50 eV and the step size was 0.05 eV. Si2p-XPS spectra were collected between 95 and 115 eV. The number of cumulative measurements was three. During the XPS measurement, a low-speed electron beam and an Ar ion beam were irradiated on the measured samples to neutralize their charges. All spectra were charge-compensated to C1s at 284.6 eV, which corresponds to hydrocarbons and a shoulder component, probably from oxidized hydrocarbons^28^.

### Surface topography

The removal spots of the RS-SiC etched by AP-PCVM were observed using a scanning white-light interferometer (SWLI, ZYGO, NewView 200CHR) and a scanning electron microscope (SEM, Hitachi S-4800). In addition, the raster scanning of RS-SiC surfaces etched by AP-PCVM with different oxygen fractions was performed by atomic force microscopy (AFM) in the tapping mode.
